# Supplementary material for: MED28 Over-Expression Shortens the Cell Cycle and Induces Genomic Instability
Source: Int J Mol Sci. 2019 Apr 9;20(7):1746. doi: 10.3390/ijms20071746 (PMC6479353; doi:10.3390/ijms20071746)
Supplement: Supplementary file 1 [file ijms-20-01746-s001.pdf]

Supplementary Information

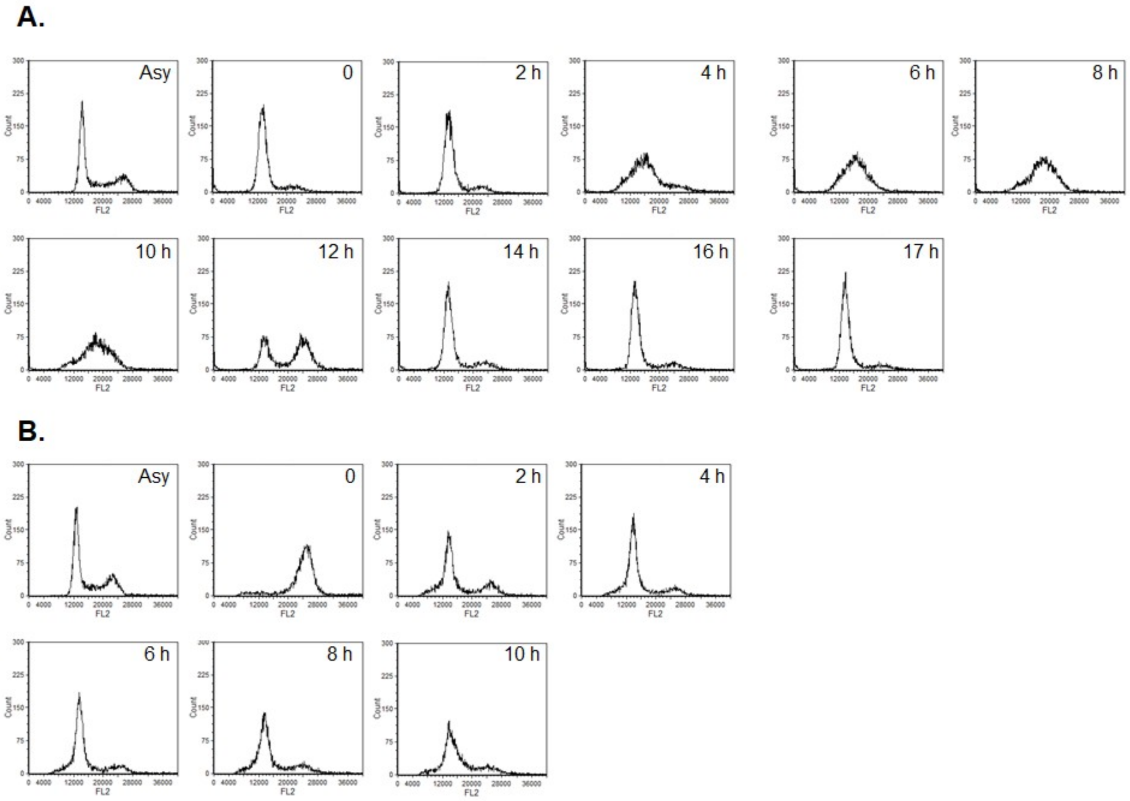

Supplementary Figure S1. Cell cycle analysis using double thymidine block or thymidine-nocodazole block. (A) HeLa cells were synchronized at the G1−S boundary by double thymidine (2 mM) block, and then released from arrest for the indicated times and analyzed by fluorescence activated cell sorting (FACS). Asy, asynchronized. (B) HeLa cells were arrested at the G2−M boundary by treatment with thymidine (2 mM) and nocodazole (100 ng/mL) and released from arrest. Cells were harvested at the indicated time points for FACS analysis. Asy, asynchronized.

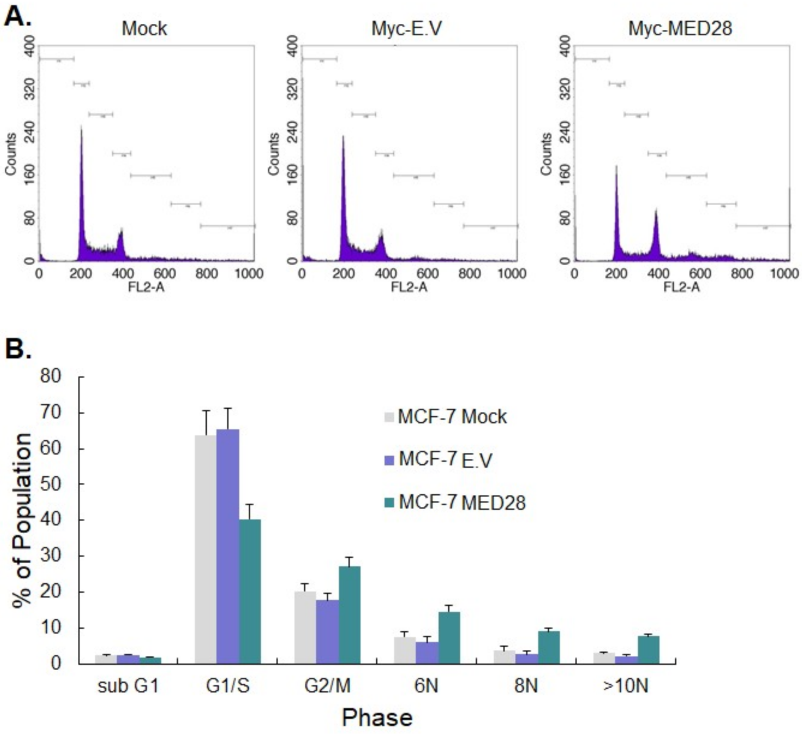

Supplementary Figure S2. Cell cycle analysis using MCF-7 cells. MCF-7 cells were transfected with pcDNA3 (E.V) or pcDNA3-mycMED28 for 36 h and selected with G418 (200 μg/mL) for 10 d. Cells were maintained in Dulbecco's modified Eagles medium (DMEM) with 10 % fetal bovine serum (FBS) and 1 % penicillin/streptomycin. (A) Cells were harvested, fixed in 70 % (v/v) ice-cold ethanol, and stained with 40 μg/mL propidium iodide (PI). The cell cycle was analyzed using fluorescence activated cell sorting (FACS). Results are from at least three independent experiments (n = 3). (B) Cell population with each phase is evaluated and presented.

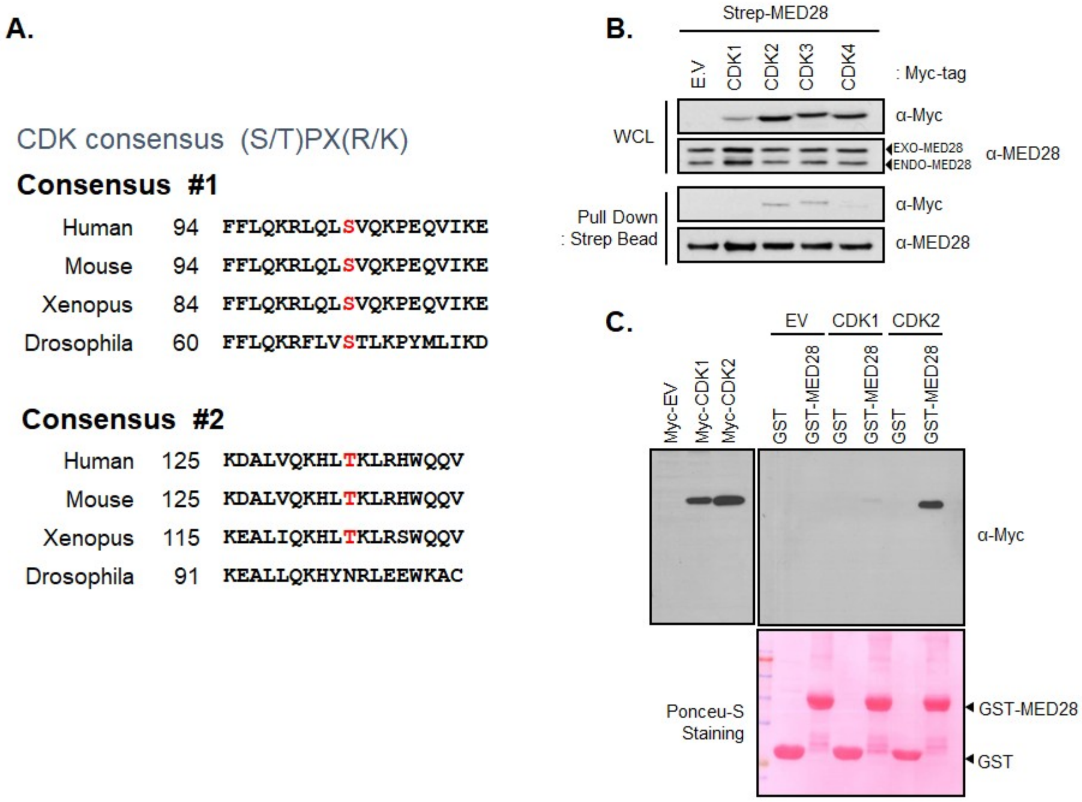

Supplementary Figure S3. Interaction of MED28 with cyclin-dependent kinase (CDK). (A) The amino acid sequence analysis of CDK phosphorylation site of MED28. (B) HEK293 cells were transfected with pEXPR-IBA103-MED28 plasmid in the presence of myc-CDK1, myc-CDK2, myc-CDK3 or myc-CDK4 for 24 h, and whole cell lysates were subjected to pull-down assay using STrEP-tactin sepharose beads to examine the interaction of MED28 with CDK. Precipitates were subjected to sodium dodecyl sulfate polyacrylamide gel electrophoresis (SDS-PAGE) for western blot. WCL, whole cell lysates. (C) Glutathione S transferase (GST) or GST-MED28 was purified as described in the materials and methods section. HEK293 cell lysates were incubated with GST or GST-MED28 for 2 h at 4 °C and washed 3 times to remove non-specific proteins. Precipitates were then subjected to SDS-PAGE for immunoblot. Ponceau- S staining was carried out to examine the equal loading and transfer of GST and GST-MED28 protein.

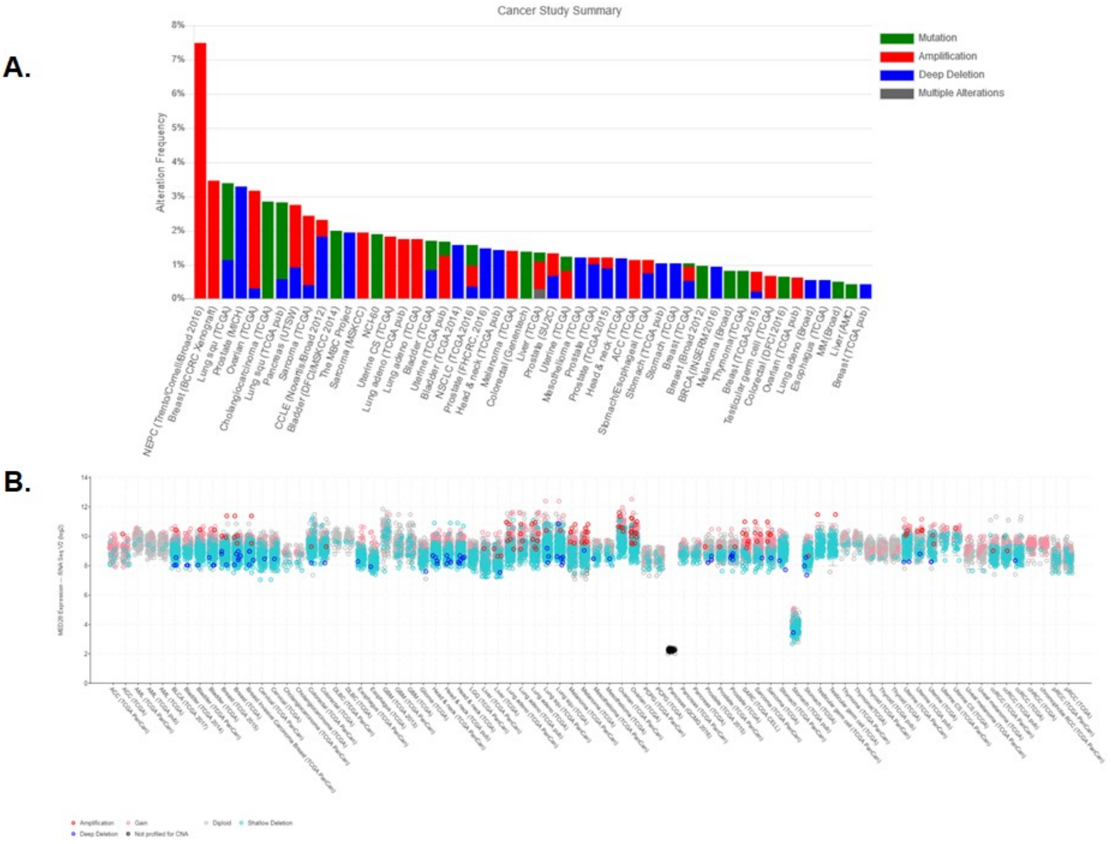

Supplementary Figure S4. (A) Cancer genome data were available on cBioPortal, an open platform for cancer genome analysis and visualization. All available cancer genome studies were used to screen Med28 mutation in different cancer type, and visualized by cancer study type. Mutation, amplification, deep deletion and multiple alterations were presented (1). (B) To present association of Med28 copy number alteration with Med28 gene expression, RNA-seq data was used and presented by TPM (log2 scale). All data was analyzed and results were visualized under cBioPortal (2). .
